# Mapping the impact of exposure to maternal immune activation on juvenile Wistar rat brain macro- and microstructure during early post-natal development

**DOI:** 10.1177/2398212819883086

**Published:** 2019-11-04

**Authors:** Tobias C. Wood, Michelle E. Edye, Michael K. Harte, Joanna C. Neill, Eric P. Prinssen, Anthony C. Vernon

**Affiliations:** 1 Centre for Neuroimaging Sciences, Department of Neuroimaging, Institute of Psychiatry, Psychology and Neuroscience, King’s College London, London, UK; 2 Division of Pharmacy and Optometry, School of Health Sciences, Faculty of Medicine, Biology and Health, University of Manchester, Manchester, UK; 3 Roche Innovation Centre Basel, Grenzacherstrasse, Switzerland; 4 Maurice Wohl Clinical Neuroscience Institute, Department of Basic and Clinical Neuroscience, Institute of Psychiatry, Psychology and Neuroscience, King’s College London, London, UK; 5 MRC Centre for Neurodevelopmental Disorders, Guy’s Hospital Campus, King’s College London, London, UK

**Keywords:** Maternal immune activation, polyriboinosinic-polyribocytidylic acid, magnetic resonance imaging, diagonal domain, rat

## Abstract

Maternal immune activation is consistently associated with elevated risk for multiple psychiatric disorders in the affected offspring. Related to this, an important goal of our work is to explore the impact of maternal immune activation effects across the lifespan. In this context, we recently reported the effects of polyriboinosinic-polyribocytidylic acid–induced maternal immune activation at gestational day 15, immediately prior to birth, at gestational day 21 and again at post-natal day 21, providing a systematic assessment of plasma interleukin 6, body temperature and weight alterations in pregnant rats and preliminary evidence for gross morphological changes and microglial neuropathology in both male and female offsprings at these time points. Here, we sought to complement and extend these data by characterising in more detail the mesoscale impact of gestational polyriboinosinic-polyribocytidylic acid exposure at gestational day 15 on the neuroanatomy of the juvenile (post-natal day 21) rat brain using high-resolution, ex vivo anatomical magnetic resonance imaging in combination with atlas-based segmentation. Our preliminary data suggest subtle neuroanatomical effects of gestational polyriboinosinic-polyribocytidylic acid exposure (n = 10) relative to saline controls (n = 10) at this time-point. Specifically, we found an increase in the relative volume of the diagonal domain in polyriboinosinic-polyribocytidylic acid offspring (p < 0.01 uncorrected), which just failed to pass stringent multiple comparisons correction (actual q = 0.07). No statistically significant microstructural alterations were detectable using diffusion tensor imaging. Further studies are required to map the proximal effects of maternal immune activation on the developing rodent brain from foetal to early post-natal life and confirm our findings herein.

## Introduction

Epidemiological studies report associations between exposure to maternal immune activation (MIA) and increased risk for psychiatric illnesses, including schizophrenia (SZ) and autism spectrum disorder (ASD) in the affected offspring (Brown and Patterson, 2011; Brown et al., 2009). Based on these findings, reverse-translational animal models of MIA were developed, particularly (but not exclusively) using the viral mimetic polyriboinosinic-polyribocytidylic acid (poly(I:C)) (Knuesel et al., 2014; Meyer, 2014). Administration of poly(I:C) on specific gestational days (GDs; range 9–17) induces a discrete inflammatory response in the dam and elicits behavioural and neurochemical abnormalities in the offspring that are of relevance for the aforementioned psychiatric illnesses, providing both causation for the epidemiological data and important mechanistic insights (Bilbo et al., 2018; Brown and Meyer, 2018; Meyer, 2014; Meyer et al., 2009). However, differences with regard to the sex, strain of rodent, dose and route of administration of poly(I:C) used to induce MIA are present in the literature (Brown and Meyer, 2018; Careaga et al., 2018; Kentner et al., 2018; Mueller et al., 2018; Smolders et al., 2018). These methodological differences likely explain the variance in the published data with regard to behavioural and post-mortem brain phenotypes in rodent MIA models (Careaga et al., 2018; Estes and Mcallister, 2016; Kentner et al., 2018; Smolders et al., 2018). Reporting guidelines and painstaking methodological work to establish the sources of variation in rodent MIA models, such as the caging system used to house the animals, are critical steps forward to address this issue (Kentner et al., 2018; Mueller et al., 2018). There is also a clear unmet need for an early outcome measure with which MIA-exposed offspring that will develop a robust behavioural phenotype may be identified to further enhance reproducibility across laboratories (Estes and Mcallister, 2016).

To address this gap, small animal magnetic resonance imaging (MRI) is one experimental approach, which offers several advantages. First, neuroanatomical phenotypes defined using high-resolution MRI in combination with advanced image processing techniques are quite robust (Lerch et al., 2012, van Eede et al., 2013). Second, MRI provides whole brain coverage, eliminating the need for a priori hypotheses concerning implicated brain regions (Finlay et al., 2014). Third, MRI operates at mesoscopic resolution, which is technically translatable to human studies (Vernon et al., 2011). This is relevant in light of recent work examining the influence of maternal cytokine levels on brain structure and function in human offspring, in relation to the development of psychopathology (Graham et al., 2018; Rudolph et al., 2018). Fourth, non-invasive MRI in rodent models may be directly linked with behavioural assays and invasive post-mortem follow-up, to establish the neural correlates of behaviour and the underlying mechanism(s) (Vernon et al., 2014). The utility of this approach is exemplified by a number of landmark studies demonstrating that induction of MIA in rodents using a range of protocols is associated with deviations from the normative developmental trajectory of brain structure, function and neurochemistry (Crum et al., 2017; Drazanova et al., 2018; Hadar et al., 2015; Malkova et al., 2014; Piontkewitz et al., 2011a; Richetto et al., 2017; Vernon et al., 2015). As yet however, a reproducible non-invasive MRI biomarker measured in early life that is predictive of later dysfunction in MIA-exposed adult offspring is yet to emerge.

Related to this, an important goal of our work is to explore the developmental trajectory of MIA effects across the lifespan (Murray et al., 2019). We have recently begun this process by exploring the effects of MIA prior to birth, at GD21 and just prior to weaning, at post-natal day (PD) 21 (Murray et al., 2019). This initial study (see also commentary by (Roderick and Kentner, 2019) provided a robust and systematic assessment of plasma interleukin 6 (IL-6), body temperature and weight alterations in pregnant rats following poly(I:C) exposure and preliminary evidence for gross morphological changes (e.g. brain-to-body weight ratio, brain weight) and neuropathology (Iba1 + microglia number and morphology) in male and female offsprings at GD21 and PD21. The relevance of these changes is currently being confirmed by behavioural assessment (Murray et al., 2019). Emerging evidence however, shows behavioural changes of relevance to psychiatric disorders in GD15 poly(I:C)-exposed offspring, including an increase in ultra-sonic vocalisation in early life (PD6) and a deficit in sustained attention in adulthood (PD125) (Potter et al., 2018). Here, we sought to complement and extend these initial results by characterising the impact of MIA on brain volume and microstructure using high-resolution, ex vivo anatomical MRI specifically at PD21. The rationale for this approach is twofold. First, it will provide a detailed, brain-wide assessment of early neuroanatomical differences between control and MIA-exposed offspring, using the protocol reported in Murray et al. (2019). Second, it will provide preliminary data (i.e. effect sizes) to inform the design of future longitudinal in vivo MRI studies, which are necessary to establish if early MRI-detectable brain changes have any functional relevance.

## Methods

### Animals

Animals used in this study were generated at the University of Manchester. Adult female Wistar rats (Charles River Laboratories, UK) were used for MIA during pregnancy. In pregnancy, rats were housed in pairs or threes before being singly housed from GD19. Rats were housed in individually ventilated cages (IVCs) with two levels (GR1800 Double-Decker Cage, Tecniplast, UK) under a standard 12- h light:dark cycle (lights on 7:00 am). The environment was maintained at 21°C ± 2°C, 55%±5% humidity. Animals had ad lib access to standard rat chow (Special Diet Services, UK) and water (Murray et al., 2019). All procedures in this study were carried out in accordance with the UK Home Office, Animals (Scientific Procedures) Act 1986 and EU Directive 2010/63/EU. The University of Manchester Animal Welfare and Ethical Review Body (AWERB) approved all experimental protocols used in this study.

### MIA and allocation of offspring for ex vivo MRI phenotyping

Induction of MIA was performed at the University of Manchester as described in detail elsewhere (Murray et al., 2019). In brief, pregnant female Wistar rats were mated at 3 months of age and GD1 confirmed by the appearance of a vaginal plug. Several studies provide evidence that poly(I:C) treatment at GD15 in rats induces a maternal inflammatory response with development of relevant behavioural phenotype(s) in the affected offspring (Mattei et al., 2014; Piontkewitz et al., 2011a; Vernon et al., 2015; Wolff and Bilkey, 2010). Pregnant Wistar rats (293.0–428.7 g) therefore received a single intraperitoneal (i.p.) injection of either poly(I:C) (n = 8; P9582, potassium salt, Sigma-Aldrich; Gillingham, Dorset, UK) at a dose of 10 mg/kg or 0.9% non-pyrogenic sterile saline as a control (n = 8) on GD15. On PD1, pups were sex typed based on anogenital distance and then culled to litters of n = 4 males and n = 4 females, which were assigned to multiple experiments running in parallel. For this specific MRI sub-study, a subset of these offspring were sacrificed at PD21 comprising n = 1 male and n = 1 female offspring from five (out of the available eight) control and poly(I:C) litters, giving a total of n = 10 (5 males and 5 females) offspring per experimental arm (control vs poly(I:C)). In the absence of data concerning effects of MIA on brain volume in Wistar rats at PD21, a formal estimation of sample size by power calculation was not possible. Rather, this study was designed pragmatically to generate this data using the minimum number of animals, in line with UK and EU guidelines (see www.nc3rs.org.uk). We therefore selected a group size of n = 10, comprising of 5 males and 5 females, such that both experimental groups were matched for sex, since the majority of studies on the effects of MIA have been predominantly carried out in male offspring only (Coiro and Pollak, 2019). This group size (n = 10) is however comparable with prior in vivo neuroimaging studies in rodent MIA models (Crum et al., 2017; Piontkewitz et al., 2011a, 2011b; Richetto et al., 2017; Vernon et al., 2015).

### Enzyme-linked immunosorbent assay

To confirm successful MIA in the dams, IL-6 concentrations in maternal blood plasma (3 h post-injection) were determined using a rat-specific enzyme-linked immunosorbent assay (ELISA) DuoSet (R&D Systems, Abingdon, UK) as reported elsewhere (Murray et al., 2019). In brief, absorbances were measured using a plate reader (MRX, Dynatech, UK) at room temperature and results were calculated from the standard curve using Prism software (v6.0, GraphPad, La Jolla, CA, USA). We present here only the IL-6 values (pg/mL) for the specific dams from which offspring were selected for inclusion in the MRI study.

### Tissue preparation for ex vivo MRI

At PD21, offspring were culled by cardiac perfusion (0.9% saline followed by 4% paraformaldehyde (PFA)) under terminal anaesthesia (sodium pentobarbital, 60 mg/kg i.p.) and the brain tissue prepared for ex vivo MRI as described elsewhere (Vernon et al., 2011). In brief, fixed brain tissues were kept intact in the cranium and post-fixed for 24 h in 4% PFA. Samples were then placed in 0.01 M phosphate buffer containing 0.05% (w/v) sodium azide to allow tissue re-hydration prior to MRI. Samples were then shipped to King’s College London (KCL) and stored at 4°C in this solution for 4 weeks prior to MRI.

### MR image acquisition

A 7T horizontal small bore magnet (Agilent Technologies Inc., Santa Clara, CA, USA) and a quadrature volume radiofrequency coil (39 mm internal diameter, Rapid Biomedical GmbH, Germany) were used for all MRI acquisitions. Fixed brain samples were placed securely one at a time in a custom-made MR-compatible holder and immersed in proton-free susceptibility matching fluid (FluorinertTM FC-70; Sigma–Aldrich, UK). Samples were scanned in a random order, with the KCL operator (ACV) blinded to treatment group (saline controls (CON) or poly(I:C) (POL) by numerical coding of samples. Scanning was interspersed with phantoms to ensure consistent operation of the scanner. Two sets of MR images were acquired: a three-dimensional (3D) Fast-Spin Echo (FSE) for structural analysis and a diffusion tensor imaging (DTI) protocol for microstructural analysis. The T2-weighted 3D FSE image had the following parameters: echo time (TE)/repetition time (TR) = 60/2000 ms, echo train length = 8, matrix size = 192 × 128 × 192 and field of view (FOV) = 28.8 × 19.2 × 28.8 mm, yielding isotropic voxels of 150 µm^3^. Total scan time was 1 h 44 min. The DTI scans were acquired using a four shot echo planar imaging (EPI) sequence with TE/TR = 35/4000 ms, matrix size = 128 × 96 and FOV = 25.6 × 19.2 mm, with an in-plane resolution of 200 µm across 50, 0.5 mm-thick slices. A total of 30 non-collinear diffusion directions were acquired, with four *b* = 0 images with a target *b* value of 2000 s/mm. Total scan time was 1 h 22 min. Reversed phase-encoded direction DTI images were additionally acquired to correct for eddy current distortions (8 min). Total scan duration for the entire protocol was 3 h 14 min per brain.

### MR image processing

After visual inspection of all MR images and elimination of those scans with artefacts, the final n values per group for statistical comparisons were: volume; CON, n = 9 (4 males and 5 females) versus POL, n = 10 (5 males and 5 females); DTI: CON, versus POL, n = 10 (5 males and 5 females). The MR images were converted to Neuroimaging Informatics Technology Initiative (NIFTI) format and processed using a combination of FMRIB Software Library (FSL) (Jenkinson et al., 2012), Advanced Normalization Tools (ANTs) (Avants et al., 2011) and in-house C++ software utilising the Insight Toolkit (ITK) library, available from https://github.com/spinicist/QUIT. The processing pipeline consisted of several steps, as described elsewhere (Doostdar et al., 2019; Wood et al., 2016). The following operations were carried out in the native space of the acquired MR images. First, a Tukey filter was applied to the FSE images in k-space to remove high frequency noise and they were corrected for intensity inhomogeneity using the N4 algorithm (Tustison et al., 2010). Second, FSL *topup* and *eddy* were used to remove distortion and eddy current artefacts in the raw diffusion data (Andersson et al., 2003; Andersson and Sotiropoulos, 2016) using acquired DTI data with a reversed phase-encode direction (see section ‘MR image acquisition’). The DTI parameter maps were then calculated using FSL *dtifit* and consisted of fractional anisotropy (FA) and mean diffusivity (MD) (Wood et al., 2016). Third, a template image was constructed from the 3D FSE images of all subjects in the study using the *antsMultivariateTemplateConstruction2.sh* script with cross-correlation metric and SyN transform (n = 19). This template was then registered to an atlas image of the PD18 Wistar rat brain, again using a cross-correlation metric and SyN transform (Calabrese et al., 2013). Fourth, all subjects FSE images were non-linearly registered to the study template using the *antsRegistrationSyN.sh* script. Logarithmic Jacobian determinants were calculated from the inverse warp fields in standard space to estimate apparent volume change, and smoothed with a Gaussian filter at a full-width half-maximum (FWHM) of 200 µm (Cox, 1996). Fifth, each subject’s DTI image was registered to the same subject’s FSE image using a SyN transform to account for residual distortions and a mutual information metric to account for the different contrast. This transform was then concatenated with those from the FSE images to the templates to align them to the study template (Wood et al., 2016). The DTI images in the template space were also smoothed with a Gaussian filter with FWHM of 200 µm. Sixth, a brain parenchyma mask was created from the atlas labels by excluding cerebrospinal fluid (CSF) regions. The inverse transforms from the atlas to the study template and from the study template to each subject were applied to calculate the brain and atlas-based region of interest (ROI) volumes for each subject (Wood et al., 2016).

### Statistical analysis

A subset of maternal plasma levels of IL-6, consisting of those values only from dams whose offsprings were selected for MRI (n = 5 CON dams vs n = 5 POL dams) were compared using unpaired t-test (2-tailed), with α = 0.05, using Prism software (v6.0; GraphPad Inc., La Jolla, CA, USA). For the MR image analysis, group-level differences in volume, FA (unit less) and MD (mm^2^ s^−1^) were assessed using atlas-based segmentation (ABS) (Crum et al., 2017), taking advantage of a publicly available high-resolution MRI atlas of the Wistar rat brain at PD18 that is parcellated into 26 regions of interest (ROIs) (Calabrese et al., 2013). We analysed both absolute (mm^3^) and relative volumes (the latter expressed as a percentage of total brain volume) to account for inter-animal variation in global brain volume affecting the volume of individual brain structures (Lerch et al., 2012; Ma et al., 2019). The FA and MD values calculated from the DTI data set were analysed without correction for total brain volume. After image registration we successfully extracted values for volume, FA and MD for 24/26 ROIs (missing ROIs: pineal gland and pituitary gland). Total brain volume was calculated from the summation of each individual atlas ROI volumes (Crum et al., 2017). Group-level differences between CON and POL-exposed animals in volumes (absolute and relative), FA and MD were assessed across these 24 ROI using 2-tailed t-test using Prism software (v8.0; GraphPad Inc., La Jolla, CA, USA) with α = 0.05. The resulting p-values from these contrasts were then corrected for multiple comparisons (to account for Type I errors across the 25 ROI) using the false discovery rate (FDR) procedure, with the threshold set at 5% (q < 0.05) (Genovese et al., 2002). Therefore, in addition to p-values, we report q-values, which are FDR-adjusted p-values. Effect sizes were calculated using Glass’ delta (Δ). In our primary analysis, we focussed on group differences between CON and POL-exposed offspring, without explicitly including sex as a biological variable (SABV) due to the limited sample size. Nevertheless, to be in line with recent policy statements regarding inclusion of SABV (Clayton, 2018) and the predominance of male offspring used in MIA studies (Coiro and Pollak, 2019) we carried our a secondary, exploratory analysis to check for sex differences using 2 × 2 analysis of variance (ANOVA) with MIA as between group factor and sex as within group factor. Due to the limited sample size, we did not carry out voxel-wise, whole brain analysis.

## Results

### Maternal circulating IL-6 levels

**Figure 1.**
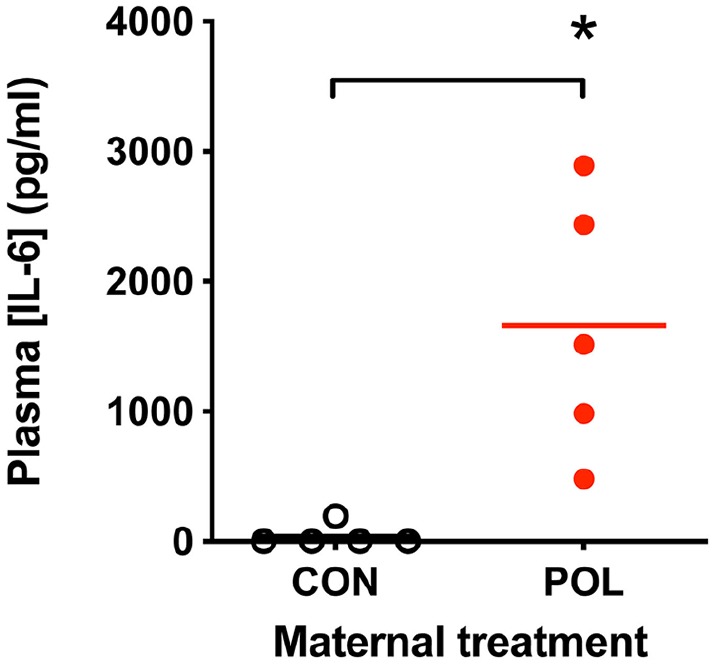
Significant increases in circulating maternal IL-6 levels 3 h post-poly(I:C) (POL) injection (plasma samples from n = 5 dams; 10 mg/kg i.p. administered on GD15) as compared to saline-injected dams (CON; plasma samples from n = 5 dams; sterile saline, i.p.) determined using ELISA-based assay. Data shown are IL-6 levels in pg/mL, *p < 0.05; 2-tailed student’s t-test.

A statistically significant increase in the circulating levels of IL-6 in maternal plasma samples could be observed 3 h post-injection in POL-injected dams as compared to controls (t = 3.62, df = 14; p = 0.007; Cohen’s d = 2.3; Figure 1). There was however, a notable degree of variability in the plasma IL-6 levels within the POL-injected dams (range 480–2890 pg/mL). No POL-exposed dams were however excluded on this basis.

### ABS analysis of volumetric and DTI datasets

#### Juvenile rat brain volume – absolute volumes

Whole brain volumes at P21 were not significantly affected by gestational POL exposure (CON: 1416 ± 37 vs POL: 1402 ± 52 mm^3^; p = 0.48; Glass’ Δ = −0.28). Of the 24 atlas ROIs compared (Table 1), the absolute volume of the diagonal domain (DD) was increased in POL offspring (+3.7%; uncorrected p = 0.03; Glass’ Δ = 1.42), although this did not survive correction for multiple comparisons (actual q = 0.78).

**Table 1.** The absolute volumes (in mm^3^) of n = 24 brain regions of interest (ROIs) in CON (n = 4 males, n = 5 females) and POL-exposed (n = 5 males, n = 5 females) offspring at PD21.

Absolute volume	CON		POL		% Diff.	p	FDR-p	Effect size
Atlas ROI	Mean	SD	Mean	SD				
Diagonal domain	4.22	0.16	4.37	0.11	3.7	0.028	0.74	1.42
Midbrain	60.60	1.42	59.25	2.18	−2.2	0.126	0.94	−0.62
Hindbrain	123.12	8.31	117.50	7.14	−4.6	0.136	0.94	−0.79
Olfactory structures	154.81	2.85	157.66	5.32	1.8	0.163	0.94	0.53
Preoptic area	7.20	0.18	7.31	0.27	1.5	0.316	0.94	0.40
Striatum	63.84	2.57	62.78	1.87	−1.7	0.325	0.94	−0.57
Internal capsule	15.67	0.44	15.45	0.63	−1.4	0.389	0.94	−0.35
Accumbens	10.06	0.40	10.19	0.35	1.4	0.436	0.94	0.39
Isocortex	486.48	10.44	480.71	21.67	−1.2	0.466	0.94	−0.27
Fimbria fornix	10.13	0.27	10.00	0.49	−1.2	0.493	0.94	−0.26
Cerebellum	189.37	11.30	185.26	18.15	−2.2	0.558	0.94	−0.23
Substantia nigra	4.51	0.15	4.46	0.24	−1.1	0.576	0.94	−0.22
Diencephalon	73.07	2.06	72.50	2.52	−0.8	0.592	0.94	−0.23
Hypothalamus	27.54	0.78	27.73	0.71	0.7	0.597	0.94	0.26
Ventricles	6.88	0.16	6.84	0.27	−0.7	0.656	0.94	−0.17
Cingulum	3.22	0.17	3.18	0.16	−1.1	0.662	0.94	−0.21
Amygdala	36.88	1.25	37.12	1.16	0.7	0.666	0.94	0.21
Hippocampus	69.74	2.05	70.17	2.71	0.6	0.700	0.94	0.16
Pallidum	10.47	0.39	10.42	0.21	−0.5	0.712	0.94	−0.26
BNST	3.31	0.18	3.30	0.17	−0.3	0.903	1.00	−0.06
Optic pathway	1.62	0.05	1.62	0.06	−0.2	0.922	1.00	−0.04
Corpus callosum	39.37	1.06	39.43	1.68	0.1	0.931	1.00	0.03
Anterior commissure	1.73	0.09	1.73	0.09	0.2	0.939	1.00	0.03
Septum	12.83	0.50	12.83	0.35	0.1	0.972	1.00	0.02

SD: standard deviation; FDR-p: false discovery rate adjusted p-value; BNST: bed nucleus of stria terminalis.

Data shown are mean ± SD. p-values are results of 2-tailed t-test (unequal variance assumed). Multiple comparisons corrections were performed using the false discovery rate procedure at 5% (FDR-p). Effect sizes were calculated using Glass’ Δ.

#### Juvenile rat brain volume – relative volumes

**Figure 2.**
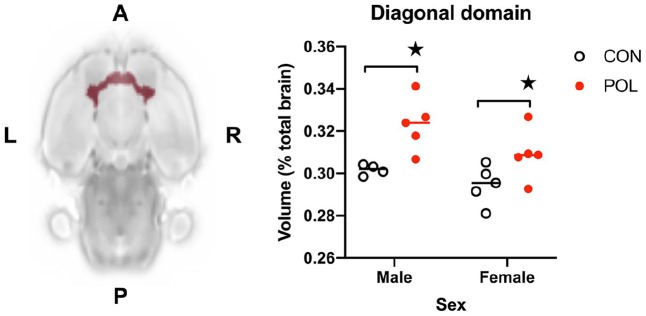
Prenatal exposure to POL at GD15 resulted in a significant main effect of MIA on the relative volume of the diagonal domain (DD) with an increase in POL-exposed offspring compared to controls at PD21. Data shown are relative volumes, expressed as the percentage of total brain volume. ★*p* < 0.01 main effect of MIA. A, Anterior; P, posterior; L, left; R, right.

We next compared relative volumes for the same atlas ROIs, which revealed 3/24 (13%) of the atlas ROIs to be affected by gestational POL exposure at an exploratory threshold of p < 0.05 uncorrected (Table 2). Consistent with the absolute volume data, the relative volume of the DD was increased in POL offspring relative to controls, with a large effect size (+6.2%; p = 0.0027; Glass’ Δ = 2.41). Nevertheless, this fell short of the required 5% FDR threshold for statistical significance (actual q = 0.07). The relative volumes of the olfactory structures and nucleus accumbens were also increased when comparing CON and POL offspring (p < 0.05 uncorrected), although again, neither effect survived 5% FDR correction (Table 2). Based on these data that consistently implicated the DD, we next carried out a secondary analysis to explore if sex differences were diluting the effect of POL exposure on DD volume. This revealed significant main effects of sex (F(1, 15) = 5.0; p < 0.05) and MIA (F(1, 15) = 14.5; p < 0.001) but no MIA × sex interaction (F(1, 15) = 0.57; n.s.) (Figure 2).

**Table 2.** The relative volumes (as a percentage of total brain volume) of n = 24 brain regions of interest (ROIs) in CON (n = 4 males, n = 5 females) and POL-exposed (n = 5 males, n = 5 females) offspring at PD21.

Relative volume	CON		POL		% Diff.	p	FDR-p	Effect size
Atlas ROI	Mean	SD	Mean	SD				
Diagonal domain	0.298	0.008	0.316	0.014	6.2	0.0027	0.07	2.41
Olfactory structures	10.933	0.279	11.394	0.553	4.2	0.0360	0.38	1.65
Accumbens	0.710	0.022	0.737	0.032	3.8	0.0488	0.38	1.21
Preoptic area	0.508	0.012	0.528	0.028	3.9	0.0603	0.38	1.71
Hypothalamus	1.944	0.038	2.004	0.101	3.1	0.1072	0.53	1.60
Amygdala	2.603	0.070	2.683	0.137	3.1	0.1278	0.53	1.13
Hippocampus	4.923	0.081	5.072	0.289	3.0	0.1473	0.53	1.83
Corpus callosum	2.780	0.059	2.849	0.157	2.5	0.2156	0.63	1.19
Septum	0.906	0.032	0.928	0.044	2.4	0.2266	0.63	0.68
Pallidum	0.739	0.015	0.753	0.035	1.9	0.2761	0.70	0.92
Anterior commissure	0.122	0.006	0.125	0.007	2.5	0.3169	0.70	0.54
Optic pathway	0.114	0.005	0.117	0.006	2.1	0.3340	0.70	0.49
Ventricles	0.486	0.009	0.494	0.029	1.7	0.4000	0.74	0.97
Diencephalon	5.160	0.164	5.240	0.282	1.6	0.4551	0.74	0.49
Hindbrain	8.683	0.369	8.497	0.683	−2.1	0.4664	0.74	−0.50
BNST	0.234	0.013	0.239	0.016	2.1	0.4679	0.74	0.39
Cingulum	0.227	0.007	0.230	0.012	1.3	0.5198	0.75	0.40
Isocortex	34.346	0.496	34.738	1.939	1.1	0.5512	0.75	0.79
Substantia nigra	0.318	0.010	0.322	0.016	1.1	0.5663	0.75	0.36
Internal capsule	1.106	0.024	1.117	0.059	0.9	0.6204	0.78	0.44
Fimbria fornix	0.716	0.027	0.723	0.052	1.1	0.6781	0.80	0.30
Striatum	4.506	0.121	4.537	0.209	0.7	0.6942	0.80	0.26
Cerebellum	13.358	0.485	13.416	1.667	0.4	0.9189	1.00	0.12
Midbrain	4.279	0.113	4.284	0.246	0.1	0.9628	1.00	0.04

BNST: bed nucleus of stria terminalis.

Data shown are mean ± SD. p-values are results of 2-tailed t-test (unequal variance assumed). Multiple comparisons corrections were performed using the false discovery rate procedure at 5% (FDR-p). Effect sizes were calculated using Glass’ Δ.

#### Juvenile rat brain microstructure (FA, MD)

There were no statistically significant effects gestational POL exposure on either FA or MD values, across any of the 24 ROIs in the Wister PD18 MRI atlas even at uncorrected p=values of 0.05 (all p > 0.05; data not shown).

## Discussion

The main findings of this study are that gestational POL exposure at GD15 results in no statistically significant neuroanatomical effects on the juvenile Wister rat brain. The DD, however, increased in relative volume by +6.2% in POL offspring with a robust effect size (Glass’ Δ = 2.42). Nonetheless, this fell just short of the accepted 5% FDR threshold for statistical significance and may therefore only be considered a trend. Additional differences in relative volumes, (p < 0.05 uncorrected for multiple comparisons), were found for the olfactory structures and nucleus accumbens, which again did not survive multiple comparisons correction. We found no significant differences, even at exploratory thresholds (p < 0.05 uncorrected for multiple comparisons), for microstructural alterations as indexed by either FA or MD. These DTI findings are however, consistent with recent data from adult Wistar Han rats exposed to POL in mid-gestation (Missault et al., 2019). Taken together, these data provide new information to suggest that there is no significant impact of the systemic POL exposure protocol as reported by Murray et al. (2019) on the juvenile Wistar rat brain at the mesoscale (Murray et al., 2019). These data may suggest that MRI-detectable structural changes in brain regions that are of particular relevance to psychiatric illness such as the prefrontal cortex and hippocampus may only emerge with increasing post-natal age following MIA exposure using this protocol. This notion is however, supported by prior MRI findings in MIA-exposed rodents (using comparable sample sizes) in which volume changes in these regions are present from PD35 onwards (Crum et al., 2017; Piontkewitz et al., 2011a). These data are also consistent with the suggested age-of-onset of hippocampus volume deficits reported in youth on the psychosis spectrum (Satterthwaite et al., 2016) and in childhood onset SZ (Nugent et al., 2007).

Limitations of this study should be noted. First, behavioural phenotyping of offspring generated using the MIA protocol used herein and reported elsewhere is currently on going (Murray et al., 2019). As such, the functional relevance of this MIA protocol still remains to be confirmed. Emerging evidence, however, suggests behavioural changes are present in this model, which have relevance to psychiatric disorders, including an increase in ultra-sonic vocalisation in early life (PD6) and a deficit in sustained attention in adulthood (PD125) (Potter et al., 2018). Nonetheless, we interpret our results only within the framework of further defining the impact of a maternal systemic poly(I:C) challenge of 10 mg/kg at GD15. Second, our sample size is small, precluding the full use of voxel-wise MR image analysis tools (Crum et al., 2017). Indeed, no findings survived a conservative multiple comparisons correction at the accepted 5% threshold. Equally, we cannot exclude the possibility that our negative results, particularly where the DTI data are concerned, are not simply a reflection of the small sample size. Arguing against this, a recent study in adult Wistar rats exposed to POL in gestation reported no microstructural changes using DTI, thus supporting our findings herein (Missault et al., 2019). Our overall group size is also comparable to previously published MRI studies in MIA models and our exploratory ANOVA analysis found no sex × group interaction. Nonetheless, we fully acknowledge that future studies in larger samples are required to investigate and confirm any sex-specific effects, as reported by others in a seminal longitudinal MRI study in another rat MIA model (Piontkewitz et al., 2011a) and to comply with the requirement to address SABV (Clayton, 2018). Third, we chose to collect ex vivo MR images, as opposed to in vivo MR images. The enhanced image quality available with ex vivo data however increases the statistical power to detect subtle volume changes when performing cross-sectional comparisons of two groups (Lerch et al., 2012; Ma et al., 2019). Balanced against this however, is the fact that the sample preparation (perfusion and tissue fixation) for ex vivo imaging may cause morphological disruption to the tissues, which could affect interpretation of the data. This is particularly true for the ventricular system, which may collapse post-perfusion, such that group-level differences in ventricular volume *in vivo* are not preserved ex vivo (Zhang et al., 2010). This is relevant as *in vivo* studies in MIA rat models do show differences in ventricular volume (Crum et al., 2017; Piontkewitz et al., 2011a). Total brain volume and that of most grey matter structures also shrinks post-perfusion (Holmes et al., 2017; Ma et al., 2019; Vernon et al., 2011). Prior work however, including our own, suggests that major group-level differences in grey matter volumes are preserved despite this shrinkage from in vivo to ex vivo and can be confirmed post-mortem (Ma et al., 2019; Vernon et al., 2011). Taken together, the choice of ex vivo MRI is consistent with the aims of this study, but the case for longitudinal in vivo studies is also reinforced. Knowledge of effect sizes from this study also allows a more precise calculation of the necessary sample size for future in vivo work. For example, calculating the Cohen’s d effect size for the DD volume increase (d = 1.4) and applying this in a power calculation suggests that a minimum of 14 offspring should be included in each group to achieve α = 0.05 with 95% power (G*Power v3.1.9.2).

Accepting these limitations, a cautious interpretation of these preliminary data suggests some interesting observations. We found no statistically significant effects (5% FDR) of maternal POL exposure on total brain volume, regional brain volumes or microstructure at PD21. Hence, robust MRI-detectable changes may only emerge with increasing post-natal age following POL exposure in this model, although this remains to be tested in depth, including in neonatal offspring (Crum et al., 2017; Guma et al., 2019; Piontkewitz et al., 2011a). At an exploratory threshold however, we found preliminary evidence for a trend towards an increase in DD volume post-MIA exposure, with a robust effect size that did not appear to be affected by sex. The DD is part of the basal forebrain, containing cholinergic, glutamatergic and gamma aminobutyric acid (GABA) neurons (Huh et al., 2010; Yoder and Pang, 2005). These neurons send efferent projections to several brain regions, in particular to the hippocampus via the medial septum (Huh et al., 2010; Yoder and Pang, 2005). During normal rodent brain development, from the third post-natal week (equivalent to our MRI window) cholinergic neurons in the DD undergo progressive hypertrophy of the soma and proximal dendrites, followed by shrinkage lasting up to the fifth post-natal week (Gould et al., 1991). Of note, a single prior study in a mouse poly(I:C) MIA model reported an increase in both the number of cholinergic neurons and the activity of choline acetyltransferase (ChAT), the enzyme responsible for the synthesis of acetylcholine in the DD of MIA-exposed offspring at both E16.5 and PD1, although later time-points were not assessed (Pratt et al., 2013). There is also evidence from human post-mortem studies for elevated ChAT activity and an increased number of cholinergic neurons in the basal forebrain of autistic individuals, but only in those aged <13 years (Kemper and Bauman, 1998), while evidence for such changes in SZ cases is lacking (Brisch et al., 2016). We tentatively suggest, based on these data, that the trend towards increased DD volume post-MIA may reflect an increase in the number of cholinergic cells and/or hypertrophy of their proximal dendrites, although this needs to be confirmed in future studies (Gould et al., 1991). Circumstantial evidence from other studies suggests this may have functional relevance. Specifically, while efferent projections from the DD innervate several brain regions, the majority project to the hippocampus via the medial septum, where they contribute to the modulation of hippocampal theta (θ) oscillations that are important for attention, spatial and working memory and sensory information processing (Gould et al., 1991; Huh et al., 2010; Yoder and Pang, 2005). It is noteworthy then that decreased θ rhythms are reported in the adult rat hippocampus following exposure to MIA at GD15, which are related to memory and sensory processing impairments in these animals (Dickerson et al., 2010; Ducharme et al., 2012; Wolff and Bilkey, 2010). Taken together, there is circumstantial evidence to suggest that elevations in DD volume following POL exposure could have functional relevance for behavioural impairments in both memory and sensory processing that are relevant for both ASD and SZ symptomatology, in line with the known associations between MIA and increased risk for these disorders. This represents a clear hypothesis for testing in future studies. In addition, future work should seek to explore whether there are any neuroanatomical effects, including in the DD following gestational POL exposure are present in the neonatal and even foetal brain, which is currently unknown (Guma et al., 2019).

## Conclusion

The findings of this study suggest no overall gross neuroanatomical remodelling of the juvenile Wistar rat brain after exposure to POL in mid-gestation (Murray et al., 2019). A trend towards increased DD volume, with a robust effect size, was however observed in offspring exposed to MIA. Longitudinal in vivo MRI studies are now required in this rat MIA model to confirm the DD volume changes, thier functional relevance with regard to adult behavioural dysfunction and the cellular basis of this effect. Such studies will also be useful for comparing the trajectory of brain volume changes in this rat MIA model with previously published data in other rat (and mouse) MIA models (Crum et al., 2017; Missault et al., 2019; Piontkewitz et al., 2011a; Richetto et al., 2017).
